# Ohio Veterinary College

**Published:** 1895-09

**Authors:** 


### AMONG THE COLLEGES.

### OHIO VETERINARY COLLEGE.

The announcement for 1895–96, states that students entering after January 1, 1896, will be compelled to enter for a three-years' course. We are sorry that this restriction could not have been enforced at the opening of the session, but will rejoice that it is to be a definite feature of the future course of the college. Thomas King, D.V.S., succeeds Dr. Neil B. Jones as professor of theory and practice of veterinary medicine. J. Francis Jones, B.Sc., V.S., M.D., succeeds Dr. Leslie A. Wright as professor of materia medica and therapeutics. Dr. Louis P. Cook, D.V.S., succeeds Dr. Francis Jones as professor of surgery. A. H. Logan, V.S., succeeds Dr. George W. Butler as professor of obstetrics and cattle pathology.

---

